# Case Report: Managing coagulopathy in neonatal acute liver failure: insights from a case of rebalanced hemostasis and thromboelastography monitoring

**DOI:** 10.3389/fped.2026.1769433

**Published:** 2026-03-27

**Authors:** Juan Huang, Mei Ye, Qiuyue Duan, Weiwei Xiao, Xiaoying Fu

**Affiliations:** Department of Laboratory Medicine, Shenzhen Children’s Hospital, Shenzhen, China

**Keywords:** acute liver failure, blood transfusion, hemostasis, neonatal coagulopathy, thromboelastography

## Abstract

**Background:**

Neonatal acute liver failure (ALF) is a rare, life-threatening condition often accompanied by complex coagulopathy. Managing this coagulopathy is challenging due to the limitations of conventional coagulation tests and the lack of pediatric-specific transfusion guidelines, often leading to empiric blood product use.

**Methods:**

We present a detailed case report of a neonate with ALF of indeterminate etiology. Clinical and laboratory monitoring included serial conventional coagulation tests (PT, APTT, INR, fibrinogen, D-dimer), coagulation factor assays, and thromboelastography (TEG). The therapeutic management and response to blood product transfusions (fresh frozen plasma, cryoprecipitate, platelets) were documented over a one-month hospitalization.

**Results:**

The patient exhibited severe and persistent abnormalities in standard coagulation parameters (prolonged PT/APTT, elevated INR, hypofibrinogenemia, and deficiency of factors II, V, VII, IX-XII, ATIII, PC, PS) alongside thrombocytopenia. In contrast, TEG revealed a normal reaction (R) time, but a reduced alpha-angle and maximum amplitude (MA). Despite 14 transfusions of fresh frozen plasma, conventional coagulation parameters showed minimal improvement.

**Conclusion:**

This case illustrates the complexity of coagulation monitoring in neonatal liver failure. It underscores the significance of comprehensive global hemostasis assays, such as TEG, which may provide superior guidance for transfusion therapy and help prevent the unnecessary administration of blood products in non-bleeding neonates with ALF.

## 1 Introduction

Acute liver failure (ALF) is a clinical syndrome caused by severe liver cell damage due to various factors including jaundice, coagulation dysfunction, hepatic encephalopathy, and ascites (1). The etiology of most pediatric acute liver failure (PALF) cases remains unclear. Common causes include neonatal hemochromatosis, herpes simplex virus infection, drugs and inherited metabolic disorders (tyrosinemia, galactosemia, and mitochondrial diseases) varies with age and geographical location. Although the incidence of pediatric acute liver failure is low, the mortality rate is high ranging from 24% to 53%, and coagulation dysfunction is one of the leading causes of death (2, 3).

The hemostatic system consists of three major arms including primary hemostasis, secondary hemostasis and fibrinolysis (2). In ALF, all three of these arms of hemostasis are affected. Platelets are responsible for the initial primary hemostatic response by adhering to the damaged blood vessels and promoting aggregation and clot formation with the aid of the endothelial-derived protein von Willebrand factor (vWF); secondary hemostasis is characterized by the formation of an insoluble fibrin clot by activated coagulation factors and thrombin, fibrin in turn stabilizes the primary platelet plug to stop the hemorrhage; fibrinolysis is the process by which the body breaks down clots. Fibrinolysis limits the extent of thrombosis, begins clot degradation, and maintains vascular patency (4).

The management of PALF is challenging due to multi-organ involvement necessitating a multi-disciplinary approach and the potential for rapid deterioration. Managing PALF is a dynamic process, with continuous monitoring. Typically, neonates have lower levels of coagulation factors and elevated screening levels at birth, these levels can be influenced by various circumstances including gestational age, labor effects, and clinical status which play into the production of coagulation factors in infants. Decreased coagulation levels lead to prolonged prothrombin time (PT) and activated partial thromboplastin time (APTT) at birth (5). Consequently, the values of screening tests in neonates and infants with ALF can be difficult to interpret and there are higher risk of poor outcomes, unnecessary correction of coagulopathy in patients without bleeding can be detrimental (6).

Here, we describe a case of neonatal ALF characterized by early onset, prolonged hospitalization, and an indeterminate etiology. Despite repeated coagulation assessments using multiple laboratory methods, along with frequent transfusions of fresh frozen plasma, suspended red blood cells, and platelets, the conventional coagulation parameters remained difficult to correct. Notably, thromboelastography (TEG) revealed a normal R value. The study provides an in-depth discussion on the complexity of this clinical scenario, the limitations of available monitoring approaches, and the therapeutic challenges encountered.

## 2 Case presentation

The patient was a female newborn delivered via cesarean section at 40 + 5 weeks of gestation, weighing 2.81 kg, with grade III meconium-stained amniotic fluid. The patient presented with hypoglycemia at birth and was subsequently admitted to the neonatal intensive care unit because of gastrointestinal bleeding. During hospitalization, the patient received fresh frozen plasma, suspended red blood cells, platelet transfusions, vitamin K1 supplementation, and hemostatic therapy, which alleviated the upper gastrointestinal bleeding. However, coagulation function tests did not return to normal levels. The patient exhibited moderate jaundice, no subcutaneous bleeding spots, petechiae, cyanosis, rash, sclerema, and no palpable superficial lymphadenopathy. Hepatosplenomegaly, elevated liver enzymes, hyperbilirubinemia, elevated alkaline phosphatase, elevated alpha-fetoprotein, abnormal coagulation parameters, and thrombocytopenia led to the diagnosis of acute liver failure. However, the cause of liver failure remains unknown.

## 3 Diagnostic and treatment process

The patient underwent a series of hematological tests, including a complete blood count, coagulation tests, and biochemical tests. During hospitalization, the patient received fresh frozen plasma(FFP) transfusions every other day, and Cryoprecipitate was administered on day 2, day 15, and day 25 of hospitalization during episodes of gastrointestinal bleeding. Hemostatic agents, such as etamsylate and vitamin K1, were also administered to prevent bleeding. Despite these interventions, coagulation abnormalities persisted. Due to generalized edema and elevated ferritin levels, it was considered likely hemochromatosis, and intravenous immunoglobulin (IVIG) therapy was administered.

Subsequently, The patient underwent exchange transfusion with the following blood products: irradiated FFP (AB-positive, Rh-positive):150 mL bag ×1, followed by 50 mL bag ×2 (total 250 mL), leukoreduced suspended red blood cells (AB-positive) 2 units, postoperatively, one therapeutic dose of platelets was administered. which improved coagulation function, liver function, and ferritin levels. However, the patient's condition relapsed with significant abdominal distension, liver failure, and uncontrolled ascites. Genetic testing did not reveal any hemochromatosis-related mutations. Tandem mass spectrometry of the blood showed elevated levels of multiple amino acids, suggesting secondary effects of liver dysfunction, although inherited metabolic disorders, such as tyrosinemia type I, could not be ruled out. Nitisinone treatment was ineffective. Despite intensive supportive care, including aggressive blood product replacement, the patient's liver function showed no significant improvement during a one-month hospitalization at our center and required transfer to a specialized facility for advanced evaluation and potential transplantation.

## 4 Results analysis

**Figure 1:**
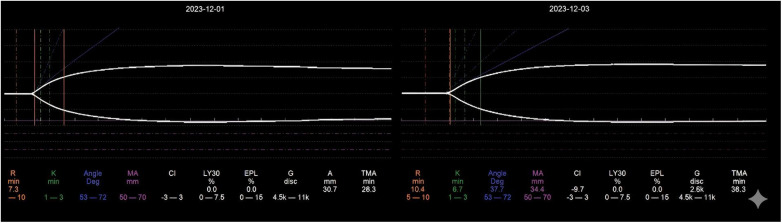
Serial TEG tracings on day 1 and day 3 of hospitalization. Upper panels show the overall viscoelastic profiles; lower tables display the corresponding numerical parameters with reference ranges. R, reaction time (min); K, kinetics time (min); Angle, α-angle (degrees); MA, maximum amplitude (mm); LY30, percent lysis at 30 min (%); EPL, estimated percent lysis (%); CI, coagulation index. Reference ranges are shown in parentheses or shaded areas.

The initial laboratory results obtained at admission revealed thrombocytopenia, prolonged APTT and PT, elevated international normalized ratio (INR), and decreased fibrinogen (Fg). Coagulation factors II,V,VII, IX, X, XI, XII, antithrombinIII (ATIII), protein S(PS), and protein C(PC), were measured two weeks after admission and found to be decreased, whereas coagulation factor VIII, von Willebrand factor (VWF), D-dimer (D-D), and fibrinogen degradation products (FDP) were increased. All coagulation assays were performed on the STA R-Evolution analyzer (Diagnostica Stago, Asnières-sur-Seine France). Initial TEG performed at admission demonstrated a decreased *α*-angle and maximum amplitude (MA), with a normal R value; however, a subsequent infection on the third day of admission resulted in a prolonged R value (Table 1, Figure 1), TEG was performed using the TEG 5,000 Hemostasis Analyzer System (Haemonetics Corporation, Boston, MA, USA) to assess whole blood viscoelastic properties,the activator is kaolin.

**Table 1:** Admission laboratory data and serial TEG parameters.

Laboratory tests	Results	Reference interval
WBC (×10^−^9/L)	12.9	6–18
HB (g/L)	106	95–183
PLT (×10^−^9/L)	49	248–586
APTT (s)	104 ↑	34.4–63.1
PT (s)	62.6 ↑	11.9–21.0
INR	7.71 ↑	0.72–1.15
TT (s)	22	14.8–22.7
FIB (g/L)	0.6 ↓	0.77–3.32
DD (μg/mL)	11.7 ↑	0.00–0.50
FDP (μg/mL)	51.4 ↑	0.0–5.0
ATⅢ (%)	10 ↓	80–120
vWF (%)	216	50–160
FⅡ (%)	13 ↓	70–120
FⅤ (%)	24 ↓	70–120
FⅦ (%)	7 ↓	60–150
FⅧ (%)	72.7	55–170
FⅨ (%)	9 ↓	60–150
FⅩ (%)	18 ↓	70–120
FXI (%)	16 ↓	60–150
FXII (%)	16 ↓	60–150
PC (%)	6 ↓	70–140
PS (%)	16 ↓	60–130
TBIL (μmol/L)	297	-——
ALP (IU/L)	1,016 ↑	51–361
AFP (ng/mL)	47,900 ↑	0–2,000
TEGday1/day3		
K (min)	5.9/6.3 ↑	1–3
Angle (deg)	40.7/37.7 ↓	53–72
MA (mm)	33.6/33.4 ↓	50–70
R (min)	7.3/10.2	5–10

**Figure 2:**
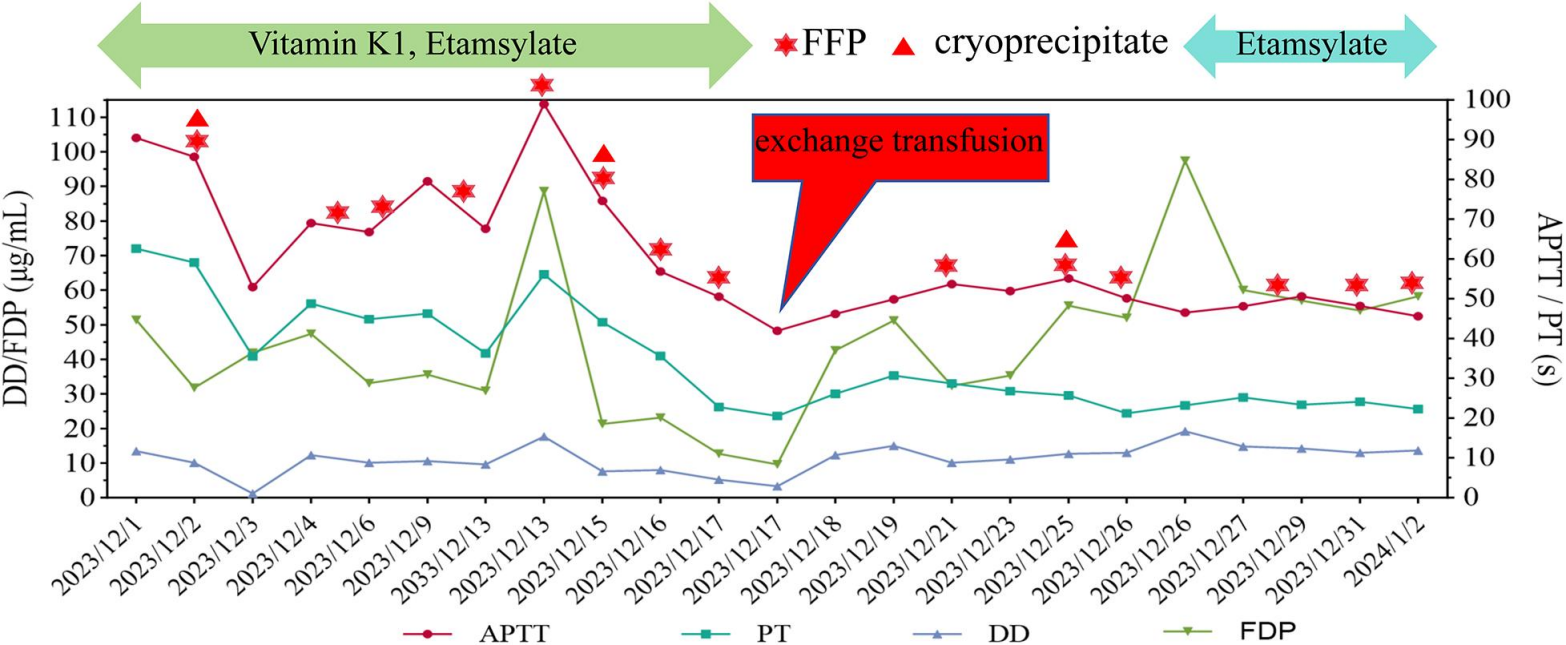
Serial laboratory data and daily records of blood product administration (fresh frozen plasma, cryoprecipitate, Vitamin K1, etamsylate) and exchange transfusion. Exchange transfusion are highlighted by a red box and arrow, indicating the corresponding laboratory and transfusion data. Dates are in year/month/day format.

Throughout the one-month hospitalization, the patient underwent extensive laboratory monitoring, including 23 routine coagulation and blood tests, 3 coagulation/anticoagulation factor analyses, and 2 TEG evaluations. FFP transfusion was administered 14 times during this period (Table 2). Despite multiple transfusions of FFP and cryoprecipitate, coagulation parameters did not significantly improve (Figure 2).

**Table 2:** Summary of key laboratory tests and treatments during hospitalization.

Project category	Specific project	Number of tests/treatments	Primary findings/purpose
Coagulation Function	PT, APTT, INR, FIB	23 times	Persistent severe abnormalities, suggesting insufficient synthesis and consumption.
Fibrinolytic Markers	D-dimer,FDP	23 times	Markedly elevated, indicating secondary hyperfibrinolysis.
Complete Blood Count	PLT, HB	23 times	Persistently low platelet count, anemia.
Overall Coagulation Assessment	TEG	2 times	Normal R value, increased K value, decreased Angle, decreased MA, suggesting poor fibrinogen function and low platelet function.
Coagulation Factor Analysis	Factors II, V, VII, VIII, IX, X, XI, XII.	3 times	Significantly reduced activity of multiple factors except for Factor VIII.
Anticoagulant System	ATIII, PC, PS	3 times	Severe deficiency in activity, indicating hepatic synthetic failure.
Key Treatment	FFP Infusion	14 times	Attempted to correct coagulation disorders, with suboptimal response.

## 5 Discussion

Almost all coagulation factors, coagulation regulatory proteins (ATIII, PS, and PC), and components of the fibrinolytic system are synthesized in the liver, while activated coagulation factors and plasminogen activators are primarily cleared by the liver (7). Therefore, in adults with severe liver disease, the absence of severe coagulopathy in some patients may be explained by a concurrent decrease in both procoagulant and anticoagulant factors. This dual impairment creates a rebalanced hemostasis (8), where the bleeding tendency induced by reduced procoagulant factors is partially offset by a concomitant loss of anticoagulant drivers. Currently, there are no accurate or efficient indicators for predicting bleeding or thrombosis in patients with liver failure (3), and no transfusion guidelines are available for PALF, which leads to the substantial use of prophylactic blood products. In adults, FFP transfusion is advised to maintain an INR between 5 and 7; however, in a more generalized pediatric population, there are age-related differences in the hemostatic profile, and there are no current pediatric cutoff values for the use of prophylactic transfusions of plasma (9). The PT and INR are measures of pro-coagulant factors and do not take into consideration changes in anti-coagulant factors, platelets, and other hemostatic changes (10). Relying solely on conventional laboratory tests to guide transfusions will lead to patients being exposed to unnecessary blood products, increasing the risk of thrombosis.

In this study, abnormal PT is closely related to the severity of liver cell damage, bleeding, and the overall prognosis. Most studies have suggested that PT is a better predictor of liver damage and clinical outcomes than bilirubin, transaminase, or albumin levels (11). The plasma fibrinogen levels in liver disease can be normal, increased, or decreased. Decreased levels of vitamin K-dependent coagulation factors (II, VII, IX, and X) reflect reduced liver synthesis capacity. Coagulation factor VIII and VWF may increase in acute hepatitis and fulminant liver failure, possibly as a response to acute inflammation, besides, it has been postulated that an increased concentration of factor VIII is due to increased expression of vWF in sinusoidal endothelial cell in liver disease, resulting in decreased factor VIII clearance (12). Thrombocytopenia may be related to reduced thrombopoietin production owing to decreased hepatocytes and increased platelet sequestration in the spleen (13). The activities of ATIII, PC, and PS are all severely deficient. These three important anticoagulant proteins are synthesized by the liver. Their extreme deficiency not only exacerbates the imbalance between coagulation and anticoagulation, leading to an increased risk of thrombosis, but also serves as a direct indicator of severely impaired liver synthetic function.

The relationship between liver failure and disseminated intravascular coagulation(DIC) is complex and variable, and requires multiple indicators for diagnosis. Studies suggest that many patients with liver disease have normal fibrinolytic potential owing to simultaneous changes in profibrinolytic and antifibrinolytic proteins. In this case, the elevated D-D and FDP levels were not significant and remained stable. In liver disease with DIC, the factor VIII activity is typically less than 50%. Therefore, the findings in this case likely reflect a state of enhanced fibrinolysis rather than a definitive DIC (14).

As a type of viscoelastic assay (VEA), TEG provides an overall assessment of hemostasis, including contributions from plasma and cellular components, while the standard tests of coagulation. In this study, the patient exhibited normal TEG R values despite abnormalities in standard lab assays such as INR. The R-value primarily reflects the speed of coagulation initiation and is most strongly influenced by factors of the intrinsic/extrinsic pathways, Within this context, factor VIII plays a critical role. In this case, the normal activity of Factor VIII perfectly explains why the R-value remains within the reference range despite such extensive deficiencies in other coagulation factors. The K-value and angle reflect the rate and strength of fibrin formation, while the MA-value mainly indicates platelet function. Abnormalities in these two sets of indicators clearly point to impaired clot formation capacity and insufficient maximum clot strength, which are associated with clinical bleeding risk (15). TEG has been shown to predict both bleeding and thrombotic events in critically ill neonates, providing more rapid and accurate information regarding the hemostatic profile and enabling more targeted therapy and rational use of blood products. Despite its clinical utility, universal standardization of TEG parameters remains limited, and robust clinical trials comparing TEG with conventional coagulation tests are scarce, available data are very limited, this limitation is especially pronounced in neonates, for whom unified normal reference values for all parameters have yet to be established. Additionally, it is important not to neglect that TEG and coagulation testing in general, may be affected by pre-analytical factors, including sampling and sample handling, as well as operator-to-operator variability (5, 16), Despite their potential utility, TEG have not yet been routinely employed in the diagnosis or management of bleeding disorders in the neonatal population. In the present study, we used kaolin-activated TEG. Notably, Mirabella et al. (17) reported no significant differences between neonates and adults with respect to kaolin-activated TEG parameters (17).

Catastrophic bleeding in PALF is rare, and most bleeding complications are clinically insignificant. Spontaneous bleeding usually due to self-limited upper gastrointestinal bleeding or post-procedural bleeding due to ICP placement (18). TEG has been shown to be superior to INR and platelet count in estimating the risk of early rebleeding. Therefore, emphasis should be placed on the role of TEG in assessing the balance between hemorrhage and thrombosis and guiding blood transfusion (19).

This case provides valuable insight into the complexity of coagulation monitoring in neonatal liver failure. The persistent and striking discordance between refractory abnormalities in conventional coagulation tests and a normal TEG R-time represents the central finding. This dissociation challenges the sole reliance on traditional parameters to guide transfusion therapy and underscores that the coagulopathy in ALF reflects a complex, rebalanced hemostatic state—a state better captured by global viscoelastic testing(such as TEG) than by factor-specific assay (20). This implies that more comprehensive assays for thrombin generation, such as the thrombin Generation Assay (TGA) and TEG may hold greater clinical value than the routine coagulation tests. William Beattie et al. (12) have demonstrated a reduced thrombin generation capacity in severe liver disease, and the TGA and thrombin dynamics capture differences in the coagulation profile that are not adequately demonstrated by the currently used coagulation tests such as INR and aPTT. The etiology of ALF in children differs from that in adults, and their procoagulant and anticoagulant factors exhibit age-specific variations. Therefore, the key to clinical management lies in a prudent evaluation of the risks and benefits of transfusion, meticulous regulation of hemostatic balance, and the effective prevention and treatment of bleeding and thrombotic complications.

## Data Availability

The original contributions presented in the study are included in the article/Supplementary Material, further inquiries can be directed to the corresponding author.
